# A Genomic Approach for Distinguishing between Recent and Ancient Admixture as Applied to Cattle

**DOI:** 10.1093/jhered/esu001

**Published:** 2014-02-07

**Authors:** Emily Jane McTavish, David M. Hillis

**Affiliations:** From the Department of Integrative Biology, University of Texas at Austin, Austin, TX 78712 (McTavish and Hillis); and the Department of Ecology and Evolutionary Biology, University of Kansas, Lawrence, KS 66045 (McTavish).

**Keywords:** *cattle*, *chromosome painting*, *hybridization*, *introgression*

## Abstract

Genomic data facilitate opportunities to track complex population histories of divergence and gene flow. We developed a metric, scaled block size (SBS), which uses the nonrecombined block size of introgressed regions of chromosomes to differentiate between recent and ancient types of admixture, and applied it to the reconstruction of admixture in cattle. Cattle are descendants of 2 independently domesticated lineages, taurine and indicine, which diverged more than 200 000 years ago. Several breeds have hybrid ancestry between these divergent lineages. Using 47 506 single-nucleotide polymorphisms, we analyzed the genomic architecture of the ancestry of 1369 individuals. We focused on 4 groups with admixed ancestry, including 2 anciently admixed African breeds (*n* = 58; *n* = 43), New World cattle of Spanish origin (*n* = 51), and known recent hybrids (*n* = 46). We estimated the ancestry of chromosomal regions for each individual and used the SBS metric to differentiate the timing of admixture among groups and among individuals within groups. By comparing SBS values of test individuals with standards with known recent hybrid ancestry, we were able to differentiate individuals of recent hybrid origin from other admixed cattle. We also estimated ancestry at the chromosomal scale. The X chromosome exhibits reduced indicine ancestry in recent hybrid, New World, and western African cattle, with virtually no evidence of indicine ancestry in New World cattle.

Geographically widespread species often exhibit considerable genetic diversity across populations. Estimating the timing and extent of divergence and gene flow among such populations is important for understanding the current structure and differentiation of individual genomes. Genomic data provide opportunities to capture the complexity of the evolutionary history of populations and reconstruct even rare historic events. Although many studies have used mitochondrial DNA (mtDNA) to study geographic variation and gene flow, the clonal maternal inheritance of mtDNA limits its usefulness (Edwards et al. 2005). Many independently segregating loci are required to capture the multiple coalescent histories that comprise a genome with hybrid ancestry (Edwards and Bensch 2009). For example, the conclusion that most humans of non-African descent have some Neanderthal ancestry (Green et al. 2010; Reich et al. 2010) would not have been possible without sufficient genomic data to capture coalescent histories that involve less than 4% of the genome. In this study, we developed a method for analyzing the structure of individual genomes to simultaneously capture information about the timing and character of admixture among groups of interacting populations.

Migration is an important evolutionary force. Gene flow among populations results in individuals that are “admixed.” The term “hybridization” is often used for admixture at the species, rather than at the population, level. However, here we are dealing with lineages near the population–species boundary and use “hybrid” and “admixed” interchangeably. Gene flow among populations can provide the genetic variation on which selection may act; conversely, admixture may swamp opportunities for local adaptation (Slatkin 1987). To make sense of the evolutionary history of populations, it is necessary to understand patterns of gene flow. Here, we explore an approach for reconstructing gene flow using genomic data, which explicitly models recombination and admixture through time. Using this approach, we can capture complex population histories and gain fine-scale information about the timing of admixture events.

Lawson et al. (2012) developed and implemented a chromosome painting model for estimating the ancestry of regions of the genome. This model has been applied to the estimation of gene flow among chimpanzee populations for conservation purposes (Bowden et al. 2012), as well as to the reconstruction of fine-scale human population structure associated with cultural differentiation (Haber et al. 2013). We extend the applications of this model to comparison of timing of admixture among populations by comparing the nonrecombined chromosomal fragment size inherited from each parent population against reference individuals for whom timing of admixture is known. Inferences about timing of admixture can distinguish between alternative phylogeographic hypotheses (Vila et al. 2005). In addition, conservation biologists can use admixture information to select appropriate candidates for conservation (Allendorf et al. 2001).

We applied this technique of estimating timing of admixture to cattle populations. A considerable database of genomic and genetic information of cattle exists as a result of the economic and environmental importance of cattle (Womack 2005). This makes cattle ideal for studying the relationships between genome architecture and hybridization. There are at least 2 major groups of domesticated cattle, which were independently domesticated from geographically disjunct populations of the wild aurochs (*Bos primigenius*) around 10 000 years ago (Loftus et al.1994). The descendants of the cattle domesticated in the Middle East are designated *B. taurus*, whereas those domesticated on the Indian subcontinent are *B. indicus*. The genome of *B. taurus* was the first assembled genome of a domesticated species (Bovine Genome Sequencing and Analysis Consortium et al. 2009; Zimin et al. 2009). The full genome sequence of *B. indicus* has also been reported and has been aligned with the *B. taurus* genome (Canavez et al. 2012). These 2 groups of cattle are more divergent than their domestication dates would suggest—a result of preexisting spatial genetic variation in the ancestral aurochs. Estimates of the age of the most recent common ancestor of all domesticated cattle range from 200 000 (Ho et al. 2008; Murray et al. 2010) to 1 000 000 years ago (Loftus et al. 1994). Nonetheless, these 2 lineages interbreed readily (Demeke et al. 2003). They are variously treated by different authors as species (*B. taurus* and *B. indicus*) or as subspecies (*B. t. taurus* and *B. t. indicus*). For simplicity and clarity, we refer to these 2 lineages as taurine cattle and indicine cattle, respectively.

Taurine and indicine cattle have some important phenotypic differences. Indicine cattle have a fatty hump at the withers, as well as a dewlap (Grigson 1991). They also have increased heat tolerance, compared with taurine cattle, and an ability to digest lower-quality forage (Cartwright 1980). Although indicine cattle are more common worldwide (Cartwright 1980), taurine cattle have been subject to more extensive artificial selection in Europe. As a result of this intense artificial selection for a number of agriculturally desirable traits (such as high meat and milk production), taurine breeds account for the vast majority of beef and dairy production, based on the numbers of registered progeny in the United States (Heaton et al. 2001).

In this study, we compare patterns of admixture among 4 groups with hybrid ancestry between taurine and indicine cattle: 1) a group composed of 2 breeds of known recent admixed ancestry dating to the early 1900s (Beefmaster and Santa Gertrudis); 2) Spanish-derived New World cattle; 3) a predominantly taurine western African breed (N’Dama); and 4) a predominantly indicine eastern African breed (Boran).

The Santa Gertrudis breed was developed from a cross of Brahman (indicine) and Shorthorn (taurine) cattle in 1918 (Rhoad 1949; Warwick 1958). Beefmaster was developed from a cross of Brahman (indicine), Shorthorn (taurine), and Hereford (taurine) cattle beginning in 1908 (Warwick 1958). Previous work (McTavish et al. 2013) has shown that Santa Gertrudis cattle have 32% (standard deviation, SD: 4%) indicine ancestry, and Beefmaster cattle have 33% (SD: 4%) indicine ancestry. Given estimates of effective generation time in cattle in the range of 2–5 years (Kidd and Cavalli-Sforza 1974; Chikhi et al. 2004), these 2 recent hybrid breeds reflect admixture within the past 20–50 generations.

African cattle have a complex history. Taurine cattle have been present in North Africa since at least 4000 before present (BP), and indicine cattle were introduced to eastern Africa by approximately 2000–3000 BP (Clutton-Brock 1999) and were present in western Africa by 1000 BP (Freeman et al. 2004). The taurine cattle in Africa may have been derived either from the same domestication as European taurine cattle or from an independent domestication of aurochs in northern Africa (Decker et al. 2009; Bollongino et al. 2012). In either case, the divergence between African and European taurine cattle is much more recent (9–15 thousand years ago (kya): Ho et al. 2008; 10–15 kya: Achilli et al. 2009; 12.5 kya: Bonfiglio et al. 2012) than the divergence between taurine and indicine cattle (84–219 kya: Ho et al. 2008; 260–300 kya: Murray et al. 2010; 335 kya: Achilli et al. 2009; 200 kya*–*1 mya: Loftus et al. 1994). Introductions of taurine and indicine cattle set up an historic cline of hybridization across Africa. This cline is marked by cattle of predominantly indicine ancestry in the east and cattle of predominantly taurine ancestry in the west, which may further be reinforced by geographically variable selection for trypanosome resistance (Loftus et al. 1994; Freeman et al. 2004). In this study, we were particularly interested in 2 African breeds: N’Dama cattle and Boran cattle from western and eastern Africa, respectively. About 32% (SD: 2%) of N’Dama genomes sampled here appear to be derived from indicine origins, as are 82% (SD: 2%) of Boran cattle genomes (McTavish et al. 2013). Some of this admixed ancestry extends into southern Europe, likely as a result of transport of cattle across the Straits of Gibraltar (Cymbron et al. 1999; Anderung et al. 2005).

New World cattle, represented here by Texas Longhorns, Corriente, and Romosinuano breeds, are the descendants of cattle brought to the New World by Spanish colonists approximately 500 years ago. These cattle also exhibit genomic signatures of admixed ancestry between African hybrid cattle and European cattle, consistent with their southern European origins (McTavish et al. 2013; Speller et al. 2013). Another possibility, however, is that some or all of the indicine genomic component found in New World breeds (11±6%) may be a result of recent introgression with indicine cattle in the New World, rather than ancient admixture (Martínez et al. 2012; McTavish et al. 2013; Speller et al. 2013). Based on variation among 19 microsatellite loci, Martínez et al. (2012) found that indicine ancestry was present in all 27 sampled New World cattle populations, but that this signal of indicine ancestry was absent in 39 cattle breeds sampled from the Iberian peninsula. Gautier and Naves (2011) also found evidence of excess African ancestry in New World cattle relative to European cattle. This pattern of African and indicine ancestry across all New World cattle may be explained by importation of admixed African cattle into the Canary Islands off western Africa; Spanish colonists used these islands as cattle depositories (Rouse 1977; Gautier and Naves 2011). These admixed cattle from the Canary Islands may have been included with Iberian cattle in the first introductions to the New World.

Here we contrast the patterns of admixture seen in cattle of ancient hybrid origin (as described above) with the patterns seen in recent taurine–indicine hybrid breeds of known origin (Santa Gertrudis and Beefmaster) and further use these differences to assess the timing of admixture in New World cattle.

The independent domestication events that led to taurine and indicine cattle captured divergent genetic information. By examining repeated instances of admixture between the 2 genomes at a range of time scales, we here examine which ancestor’s alleles have been maintained through time. In addition, we examine whether or not the genomic architecture of introgression is similar between independent origins of hybrid lineages. We also use patterns of recombination and sizes of linkage blocks to compare the ages of admixture events and further assess the evidence for recent versus ancient admixture. The scaled block size (SBS) metric that we developed can be applied to the assessment of the timing of admixture in other species also.

## Materials and methods

We analyzed 1369 individuals of 58 breeds genotyped at 54 001 single-nucleotide polymorphisms (SNPs) loci using an Illumina 55K chip (Matukumalli et al. 2009). We performed analyses on all breeds together, but we focused on the 4 groups of 7 breeds that were of particular interest to our questions, as described above. The sampling across these groups consisted of 1) recent hybrids: *n* = 46 (Beefmaster: *n* = 23; Santa Gertrudis: *n* = 23); 2) New World cattle: *n* = 51 (Texas Longhorns: *n* = 40; Corriente: *n* = 4; Romosinuano: *n* = 7); 3) western African N’Dama: *n* = 58; and 4) eastern African Boran: *n* = 43. The primary data underlying these analyses have been deposited with Dryad following data-archiving guidelines (Baker 2013).

### Filtering and Phasing

We removed the SNP loci from our analysis if 1) they were missing from the manifest and could not be decoded; 2) if average heterozygosity was greater than 0.5 in 10 or more breeds (an indication of paralogy or repeat regions); 3) if call rate was lower than 0.8 in 10 or more breeds (an indication of null alleles); or 4) if data from a given locus were missing in at least 70% of sampled individuals. We then removed individuals with greater than 10% missing data across the loci on the 29 autosomes and the X chromosome and subsequently removed loci that were missing in greater than 10% of individuals. Totally, 1369 individuals and 47 506 autosomal markers remained after filtering. The list of loci is available, along with the data, at doi:10.5061/dryad.42tr0. For the X chromosome, we also excluded the estimated pseudoautosomal region (PAR) based on the UMD3.1 genome assembly (physical map locations greater than or equal to 137 109 768bp; Zimin et al. 2009). After removal of the PAR, 872 X-linked loci remained in our analyses.

We phased the SNP loci into haplotypes and imputed missing data simultaneously using fastPHASE (Scheet and Stephens 2006). We used fastPHASE to estimate the number of haplotype clusters via a cross-validation procedure described in the study by Scheet and Stephens (2006). We did not take population of origin into account in phasing and used 20 random starts of the expectation-maximization algorithm. Pei et al. (2008) found fastPHASE to be the most accurate among available genotype imputation software. We conducted all analyses on phased data.

### Determination of Sex

Because sex was not recorded for some samples from previously collected data sets, we estimated sex from polymorphisms at markers thought to be on the X chromosome. As males only have 1 X chromosome, they are not expected to be polymorphic at X-linked loci. We excluded the PAR region of the X chromosome, as described in the section on Filtering in Materials and Methods. Based on samples of known sex, as well as the bimodality observed in plotting polymorphism on the X chromosomes across all individuals, we assigned individuals with less than 1% polymorphism at X-linked loci as males. We used the 1% threshold to account for possible genotyping error. We recoded the less than 1% of called heterozygous alleles in males as missing data. By this assignment, we had a total of 352 females and 1017 males.

### Model-Based Clustering

We performed model-based clustering analysis for each chromosome using Bayesian parametric analysis, based on a fit to the Hardy–Weinberg equilibrium model, as implemented in the software STRUCTURE (Pritchard et al. 2000). In order to differentiate histories across chromosomes, we independently analyzed each of the 29 autosomes and the X chromosome. The SNPs from each chromosome were analyzed using the linkage model based on their UMD3.1 map positions (Zimin et al. 2009). We ran the Markov chain Monte Carlo simulation for 20 000 generations and used a burn-in of 1000 generations. Recombination rate was treated as uniform. For X-linked loci in males, we used hemizygous genotypes. We ran 5 independent Markov chain Monte Carlo runs. To address the potential for bias that may result from unequal sample sizes across groups (Kalinowski 2011), we assigned our sample of 1369 individuals to 5 a priori groups (viz., indicine, taurine, African, New World, and recent agricultural hybrids) and resampled to create equal sample sizes, of 25 individuals each, across these groups. We then performed STRUCTURE analyses on these subsamples. We calculated the correlation between individual admixture proportions before and after resampling in Python using the *scipy.stats.pearsonr* function (Jones et al. 2001).

### Significance Testing

We used a bootstrap resampling approach (Efron 1981) to test for significant departures from median admixture proportions of individual chromosomes within breeds. Because distributions of proportions are not normal, we could not use methods that assume normality for these tests. We tested for significant differences across chromosomes in the median and the variation of admixture proportions, compared with the expected distributions, assuming uniform admixture across chromosomes within breeds. For these tests, we first calculated the median taurine ancestry for each chromosome for each individual. Using these values, we created a distribution of taurine ancestry consisting of all the proportions for all chromosomes for all the individuals of each breed. We then drew bootstrap samples of new chromosomes by sampling from this distribution. We then compared the actual median introgression of each chromosome in the original data with the expected distribution (if admixture were uniform across chromosomes). We performed 50 000 resampling replicates to generate the expected distribution and used a Bonferroni-corrected α-value of 0.0002 (2-tailed test). This value was calculated by taking a *P* value of 0.025 for a 2-tailed test and dividing by 120 to account for multiple tests of 30 chromosomes across 4 different groups.

To test for significant deviations in variability across chromosomes, we calculated the absolute difference from the group median for each individual for each chromosome and performed an Anova on these values (Levene 1960). As the deviations from the mean were not normally distributed, we created an expected distribution of *F*-statistics by resampling from this pool and performing an Anova on the distributions of the randomized deviations from the median (Boos and Brownie 2004). We performed 5000 resampling replicates in this test. All Anovas were performed in Python using the *scipy.stats.F_oneway* function (Jones et al. 2001). Correlations were calculated in Python using the *scipy.stats.pearsonr* function (Jones et al. 2001).

### Chromosome Painting

We used Li and Stephen’s (2003) copying model, as implemented in ChromoPainter (Lawson et al. 2012), to estimate regions of ancestry across the chromosome. This model relates the patterns of linkage disequilibrium (LD) across chromosomes to the underlying recombination process and avoids the assumption that LD must be block like by computing LD across all sites simultaneously. This method uses a Hidden Markov Model to reconstruct a sampled haplotype as it would be generated by an imperfect copying process from all other haplotypes in the population. Ancestry of regions can be inferred by estimating copying probabilities from 2 or more donor populations for chromosomal regions of admixed individuals. An estimate of “copying” from a population is equivalent to inferring that a particular region of a haplotype coalesced with that of an individual from the identified population more recently than with that of an individual of another population. Using this approach, we were able to assign ancestry of regions along chromosomes, even when there were no fixed differences among populations because the method takes into account the physical position of loci and makes estimates based on all sites simultaneously. We used an estimated effective population size for all breeds together of 4000, as estimated from the ChromoPainter software. This estimate is consistent with the low estimates (in the 100s) of effective population sizes for most European breeds of cattle (Bovine HapMap Consortium 2009).

To “paint” the admixed chromosomes with ancestry from the taurine and indicine lineages, we used representative “donor” populations of genotyped taurine and indicine cattle. The 2 donor populations (taurine and indicine) were composed of individuals that were estimated to have less than 2% of introgressed ancestry. The individuals selected by this procedure were members of breeds a priori expected to represent the taurine and indicine lineages. These donor populations consisted of 502 taurine individuals and 151 indicine individuals. Because we were interested in admixed groups, we set equal a priori probabilities of copying from either of these donor populations. Because the likelihood estimate is dependent on the order in which individual haplotypes are considered, we used the averaged estimates across 5 random runs of the expectation-maximization algorithm.

### Timing of Admixture

Baird (1995) showed that following admixture, the breakdown of linkage among alleles from parental population occurs slowly and may be used to estimate time of contact. Theoretical expectations for breakdown of linkage through time are mathematically straightforward and were described by Fisher (1954). However, genetic details such as differences in recombination rate across chromosomal regions present obstacles for making empirical estimates of time from admixture data. To obtain a metric of timing for introgression events, we calculated the scaled median introgressed block size, which we refer to as SBS. In all cases, 1 of the 2 ancestral populations made up the majority (greater than 50%) of the genome of an individual. We treated that ancestral population as the “parental” genome, and the alternate (minority) ancestor as the “introgressed” genome. SBS is calculated using the introgressed genome. For each individual and chromosome, we calculated median block size of introgressed DNA as a proportion of the chromosome (range: 0–0.5). The maximum is 0.5 because we were using the proportion of the chromosome inherited from the introgressed (minority) ancestor. We used medians rather than means because distributions were skewed. We scaled block sizes by dividing the median introgressed block size by the overall proportion of the chromosome inherited from the introgressed ancestor. We used a chromosomal scale because we were interested in inferred recombination events. If only 1 recombination event occurred since admixture, the introgressed region would be expected to lie in a single segment, and the scaled average block size would be 1. However, as further recombination and backcrossing occurs, the introgressed material is divided up across the genome, and the block size decreases. Before regions of ancestry are fixed, introgressed block size is expected to be strongly correlated with time since introgression (Baird 1995; Ungerer et al. 1998; Rieseberg et al. 2000). For each individual, we averaged values of SBS across all autosomal haplotypes.

## Results

### Model-Based Clustering

**Figure 1.**
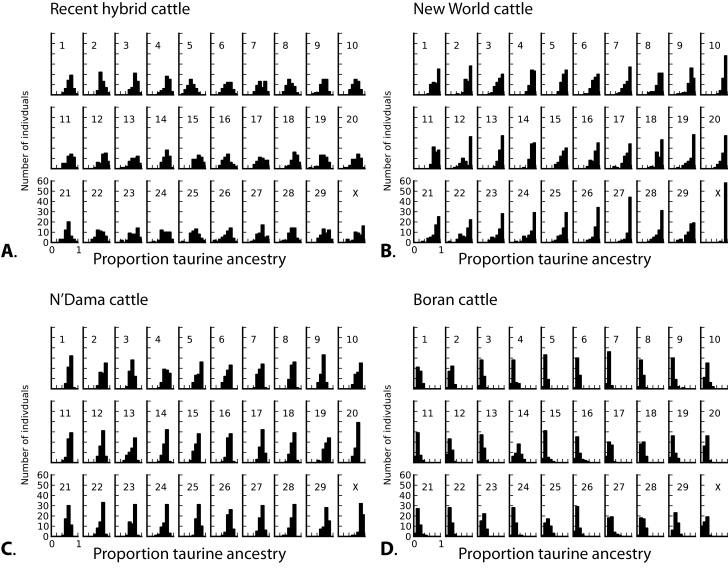
Histograms showing estimated proportion of taurine ancestry for individuals on each chromosome. The *x* axis indicates estimated taurine ancestry as calculated using STRUCTURE. The *y* axes are scaled to percentages of sampled individuals. (**A**) Recent hybrid cattle (Beefmaster and Santa Gertrudis). (**B**) New World cattle (Texas Longhorns, Corriente, and Romosinuano). (**C**) Western African cattle (N’Dama). (**D**) Eastern African cattle (Boran). Note near-complete absence of admixture on the X chromosome in New World cattle.

**Figure 2.**
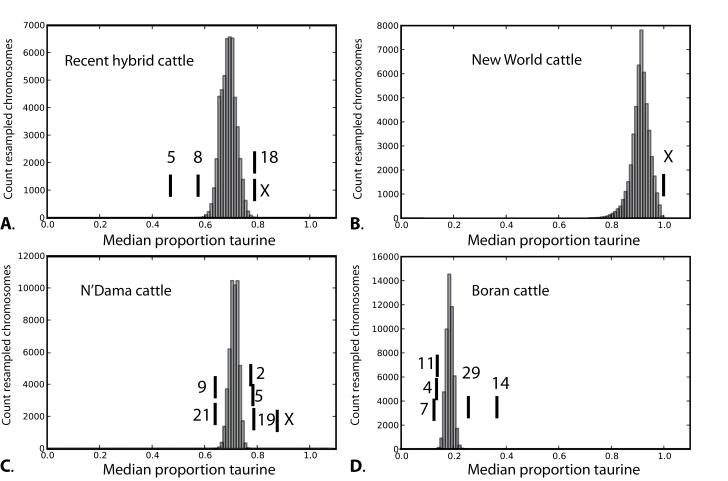
Monte Carlo resampling of median ancestry across chromosomes. Distributions show the expected median values of ancestry across chromosomes (assuming introgression is randomly allocated), and vertical lines represent the actual median values of introgression for each chromosome that is significantly different from the expected distribution (see Table 1). (**A**) Recent hybrid cattle (Beefmaster and Santa Gertrudis). (**B**) New World cattle (Texas Longhorns, Corriente, and Romosinuano). (**C**) Western African cattle (N’Dama). (**D**) Eastern African cattle (Boran).

We reconstructed the distributions of ancestry across chromosomes for individuals in each of the 4 study groups (Figure 1). We averaged admixture proportions for each individual for each chromosome across runs. All runs converged on highly congruent estimates. The maximum range of ancestry estimates for an individual across all 5 runs was 3% points. We found that several chromosomes exhibited significant differences in median introgression levels compared with expectations under a model of equal introgression across chromosomes (Table 1; Figure 2). Although no particular chromosome showed extreme patterns of introgression in all 4 groups, the X chromosome had reduced indicine ancestry in recent hybrid cattle, New World cattle, and N’Dama cattle (Table 1). This pattern was not shared with eastern African Boran cattle. We did not find any differences in variability of admixture proportions across chromosomes within groups. The correlation between estimated values of admixture proportions before and after resampling (to produce even sample sizes) was extremely strong (*r* = 0.996, *P* < 0.00001), indicating that uneven sample sizes across groups had minimal effect on our estimates of admixture proportions.

**Table 1:** Chromosomes that fall outside the expectations for distribution of taurine ancestry, assuming ancestry proportions are uniform across chromosomes

Groups	Significant chromosomes	Median proportion of taurine	Range	Lower cutoff at α = 0.0002	Upper cutoff at α = 0.0002	*P* value
Recent Hybrids	*Bootstrap sample*	*0.69*	*0.558–0.813*	*0.59*	*0.79*	—
	5	0.50	0.152–0.720	*	—	<0.00002
	8	0.58	0.320–0.857	*	—	<0.00004
	18	0.79	0.416–0.988	—	*	<0.00014
	X	0.79	0.356–0.999	—	*	<0.00014
New World cattle	*Bootstrap sample*	*0.91*	*0.647–0.995*	*0.71*	*0.99*	—
	X	1.00	0.718–0.999	—	*	<0.00002
N’Dama	*Bootstrap sample*	*0.71*	*0.632–0.771*	*0.65*	*0.76*	—
	2	0.78	0.549–0.866	—	*	<0.00002
	5	0.79	0.527–0.882	—	*	<0.00002
	9	0.64	0.435–0.726	*	—	<0.00008
	19	0.78	0.469–0.898	—	*	<0.00002
	21	0.64	0.493–0.744	*	—	<0.00008
	X	0.88	0.522–0.993	—	*	<0.00002
Boran	*Bootstrap sample*	*0.19*	*0.134–0.251*	*0.14*	*0.23*	—
	4	0.14	0.042–0.378	*	—	<0.00004
	7	0.13	0.066–0.251	*	—	<0.00002
	10	0.23	0.141–0.435	—	*	<0.00010
	11	0.14	0.064–0.373	*	—	<0.00004
	14	0.37	0.132–0.550	—	*	<0.00002
	29	0.26	0.128–0.485	—	*	<0.00002

Medians and ranges are shown for chromosomes with more extreme values than expected based on bootstrap samples, as described in text (Bonferroni-corrected *P* value = 0.0002). Asterisks (*) indicate significant deviations below the lower significant cutoff value or above the upper significant cutoff value. The values of the bootstrap samples are italicized. The bootstrap distributions and median values for outlying chromosomes are shown in Figure 2.

### Chromosome Painting

**Figure 3.**
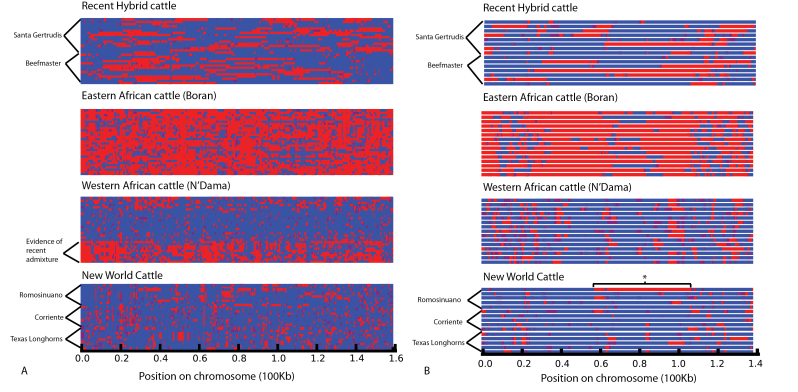
Admixed ancestry across chromosomes. Ancestry of chromosomal regions estimated by ChromoPainter. (**A**) Chromosome 1 inferred from 3150 SNP markers (**B**) X chromosome with pseudoautosomal region excluded, inferred from 872 SNP markers. Each horizontal line represents a haplotype (2 from each individual on chromosome 1; single haplotypes displayed for the X chromosome), and the colors represent estimated ancestry of each chromosomal region (blue indicates greater than 75% probability taurine; red indicates greater than 75% probability indicine; yellow indicates intermediate probabilities). The 2 donor populations (taurine and indicine) were based on individuals that were estimated to have less than 2% of introgressed ancestry. The figure illustrates 15 representative individuals from each of 4 groups of interest (New World cattle, N’Dama, Boran, and recent hybrids). The asterisks (*) mark evidence of recent introgression in a Romosinuano individual.

We reconstructed the ancestry of chromosomal regions through chromosome painting (Figure 3). This analysis indicated differences in structure of ancestry both within and between populations. As expected, large nonrecombined tracts of DNA from each ancestral linage were apparent in recent hybrid breeds, such as Beefmaster. The analysis also indicates differences among groups within breeds. N’Dama cattle showed breed substructure associated with sample identity number, shown by the label “evidence of recent admixture” in Figure 3. This suggests different population histories associated with time of sample collection and therefore herd of origin.

### Quantitative Comparisons

**Figure 4.**
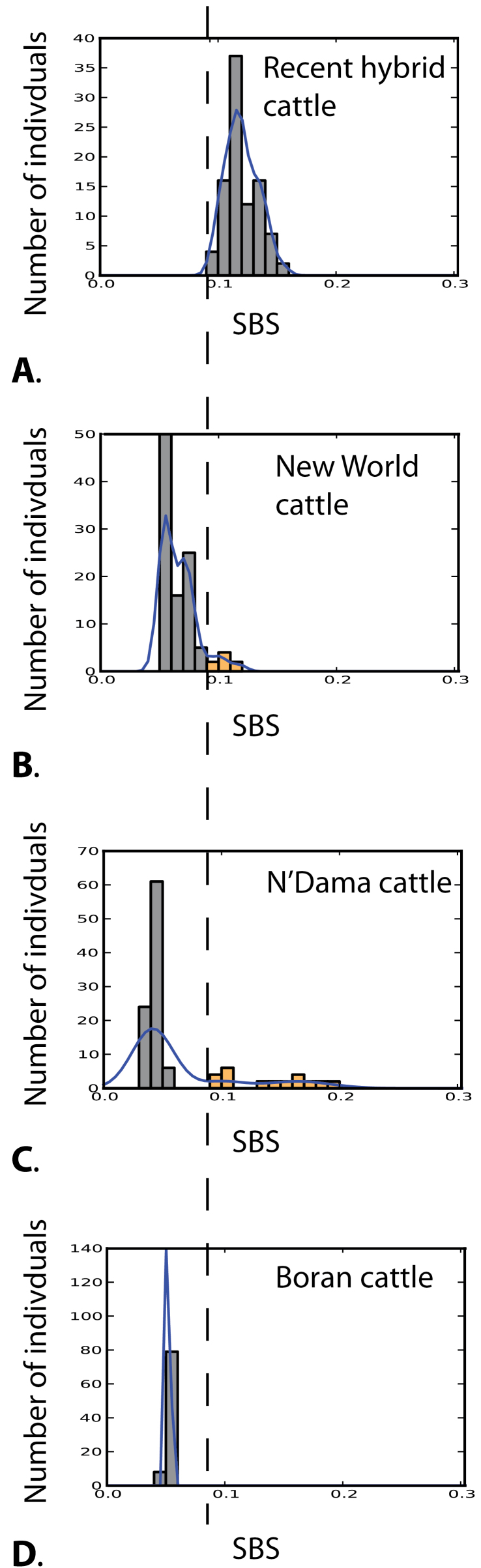
The distribution of scaled average introgressed block sizes (SBSs) of the less-common genome. Values greater than 0.09, shown by dashed vertical line, overlap with values for known recent admixed individuals. (**A**) Recent hybrid cattle (Beefmaster and Santa Gertrudis). (**B**) New World cattle (Texas Longhorns, Corriente, and Romosinuano). (**C**) Western African cattle (N’Dama). (**D**) Eastern African cattle (Boran).

**Figure 5.**
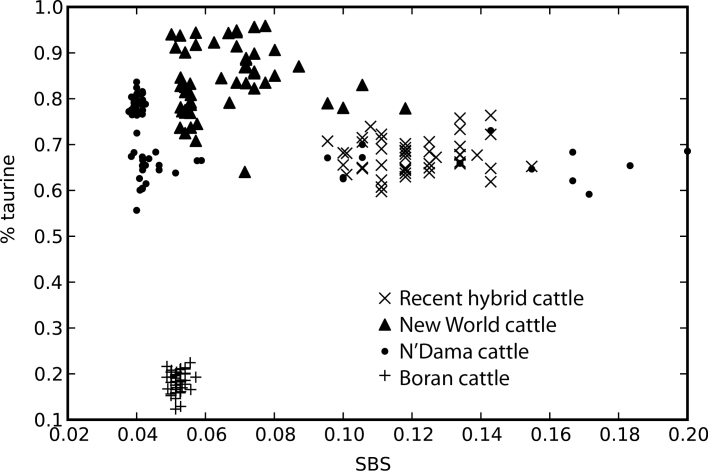
Proportion of taurine ancestry versus SBS score for individuals shown in Figure 4. Proportion of taurine ancestry has been taken from McTavish et al. (2013) and is shown plotted against SBS score.

Estimates of SBS differed across groups (one way Anova; *P* < 0.00001). We found that recent hybrid cattle have larger nonrecombined blocks of introgressed genetic material, as measured by the SBS metric, compared with New World cattle, N’Dama cattle, or Boran cattle (Figure 4). New World cattle and both African groups each had smaller introgressed fragment sizes than recent hybrid breeds, reflecting their older admixed ancestry (Figure 4). There was no significant correlation between estimated proportion of taurine ancestry and SBS score for recent hybrid cattle (*P* = 0.23), New World cattle (*P* = 0.50), or Boran cattle (*P* = 0.09), and there was a weakly negative correlation in N’Dama cattle (*r* = −0.37, *P* = 0.00004). SBS can differentiate timing of introgression even among individuals with the same overall proportion of introgression (Figure 5). Each of the groups N’Dama, Boran, and New World cattle had modal SBS close to 0.5, whereas in recent hybrid cattle, it was approximately 0.11. The minimum SBS value for an individual of known recent hybrid cattle was 0.09. Using this value as a cutoff for admixture within the past 100 years, we found a few individuals within both N’Dama and New World cattle breeds that showed evidence of relatively recent indicine introgression. These bins are shown in orange in Figure 4. An individual of New World origin with an SBS value of 0.076 also had a large nonrecombined block of indicine origin on the X chromosome (marked by an “*” in Figure 3), strongly suggesting recent admixture.

## Discussion

The similarity of scaled indicine fragment sizes in African cattle and New World Spanish-derived cattle suggests that the admixture observed between taurine and indicine lineages in New World cattle predated or was concurrent with their introduction to the New World. This pattern is consistent with the hypothesis of crossing between admixed African lineages and taurine lineages from the Iberian Peninsula in the Canary Islands (the source for at least some of the Spanish cattle imports into the New World; Rouse 1977).

Introgression becomes progressively harder to reconstruct with time. Denser genomic sampling is required to reconstruct smaller blocks of LD (Villa-Angulo et al. 2009). However, if populations are not subject to gene flow following admixture, eventually, introgressed blocks will become fixed in the population or be lost due to drift (Ungerer et al. 1998; Rieseberg et al. 2000). Through time, the variance in tract length inherited from each ancestor decreases (Gravel 2012). After introgressed regions in a population are fixed, no further information about timing of admixture can be gleaned from introgressed block size.

In addition to differences in timing of admixture among groups, we found differences among individuals within groups. Individual SBS values were unimodal and showed close-to-symmetrical distribution in Boran and recent hybrid cattle. This suggests that values were drawn from a single distribution and is consistent with a uniform admixture history within those groups. In contrast, the distributions of scaled fragment sizes appear skewed to the right in both N’Dama and New World cattle (Figure 4). The skewed distributions toward larger blocks of introgressed material in these groups are consistent with those individuals having undergone more recent admixture. We used the lowest SBS score of known recently admixed cattle as a lower cutoff to distinguish individuals of likely recent admixture. However, the SBS metric relies on scaling sizes of introgressed fragments by the overall introgressed proportion of each respective chromosome, which may limit the usefulness of this approach at very low levels of introgression. This metric can be applied to the estimation of timing of admixture in other species for which at least some known hybrid individuals have been sampled.

For these analyses, we used physical map distances from the UMD3.1 assembly of the taurine (*B. taurus*) genome (Zimin et al. 2009). Ideally, we would use genetic map distances for our chromosome painting analyses. Previous linkage maps have found concordance between physical map and genetic map locations (Arias et al. 2009), but there is currently no full linkage map for the SNP loci we analyzed. In addition, although the *B. indicus* genome has been sequenced, it was assembled through alignment with the *B. tauru*s genome. Thus, some synteny differences may have been missed. Synteny differences would affect recombination rates between these genomes and could bias estimates of absolute dates of admixture. We mitigated this bias by using comparisons among groups derived from recombination between these same 2 ancestral lineages. By comparing among groups, we can standardize for bias that results from changes in recombination rate across regions between these 2 taxa.

Estimates of absolute timing of admixture would be of interest to archeologists, breeders, and phylogeographers. However, our lack of precise knowledge of population sizes and recombination rates preclude our making those estimates. Both population size and recombination rate factor into scaling estimates to time. Large populations with low recombination can act like small populations with higher recombination rates. We do not attempt to tease apart those factors here, although with better linkage maps, it may be possible in future. However, even in studies of admixture in humans, in which recombination is well understood, distinguishing the effects of population size and recombination rate has proven difficult (Blum and Jakobsson 2011).

Haplotype-based techniques have been used recently to interrogate admixture histories in many human populations (Kim et al. 2012; Palamara and Pe’er 2013; Gravel 2012). Harris and Nielsen (2013) used variance in shared haplotype length to infer demographic parameters. However, Harris and Nielsen’s (2013) technique requires exact matches to infer segments of ancestry (identity by state). Applying the “ChromoPainter” chromosome painting model to our SNP data (Li and Stephens 2003; Lawson et al. 2012) has several advantages. Due to bias in the selection of loci used on the SNP chip (Matukumalli et al. 2009), each SNP has high minor allele frequencies and is highly polymorphic even within groups. Therefore, although our analysis included many loci, each individual locus provides limited ancestry information. The high minor allele frequencies reduce the power for methods that rely on pairwise allele sharing to estimate LD and timing of admixture, such as *rolloff* (Moorjani et al. 2011; Patterson et al. 2012). But by coestimating across all loci and using linkage information to inform our model of genomic regions of ancestry using ChromoPainter, we were able to integrate information from many sites to estimate recombination breakpoints since admixture.

Although these types of chromosomal linkage-based techniques for estimating admixture have been applied principally to primates, our results build on a large body of phylogeographic research on the history of domestication and admixture in cattle. Although the hypothesis of 2 main domestications of cattle is broadly supported by archeological as well as genomic data, there is some support for a third independent domestication of the aurochs in Africa. However, whether the European–African split dates to pre- or postdomestication, genetic data strongly support a sister-group relationship between European and African cattle, relative to Indian cattle (Murray et al. 2010). Although estimates of the timing of divergence between taurine and indicine cattle, as well as between European taurine and African taurine cattle, vary across analyses, the former is estimated to be an order of magnitude higher than the latter (Loftus et al. 1994; Ho et al. 2008; Achilli et al. 2009; Murray et al. 2010). Therefore, our chromosome painting results, as well as STRUCTURE analyses, should capture genetic differences that resulted from the deepest split within cattle (i.e., that between the indicine and taurine lineages).

Gautier et al. (2010) argued that STRUCTURE estimates at *K* = 2 (i.e., 2 assumed populations) inflate indicine ancestry in African taurine cattle, whereas at *K* = 3, that variation is absorbed into an “African-like” cluster. Gautier et al. (2010) showed that when ancestry is divided into 3 rather than 2 major clusters, the impact of indicine admixture on West African cattle is decreased. However, even with more extensive sampling across African cattle, and when using 3 clusters to reflect ancestral populations, Decker et al. (2013) found taurine–indicine admixture in most, but not all, West African cattle breeds. We found evidence of indicine introgression in all West African cattle sampled here.

Kalinowski (2011) noted that STRUCTURE analyses may be biased when estimating ancestry if the true number of groups is greater than the value of *K* used. However, this problem is most pronounced when there is a short branch in the phylogeny deep in the past. That problem is unlikely to be an issue in this case, because the divergence between the 2 major groups, taurine and indicine cattle, is much deeper than divergences within taurine cattle. Indeed, in contrast with Kalinowski’s (2011) results, we found STRUCTURE estimates of admixture to be extremely robust to changes in sample sizes based on our resampling experiments. Nonetheless, there is potential for bias in our estimates of introgression, because the ancestral taurine cattle are represented only by European taurine cattle as we do not have samples of nonadmixed African taurine cattle. The choice of donor populations that represent European taurine and Indian-subcontinent indicine lineages may decrease our power to estimate blocks of ancestry in deeply diverged African taurine lineages.

Bolormaa et al. (2011) showed that differences in ancestry of chromosomal regions were associated with significant quantitative trait loci for beef production and growth. In all groups sampled, we found at least 1 chromosome that was not consistent with a uniform distribution of introgressed ancestry across chromosomes. However, with the exception of the X chromosome, these differences were not consistent across groups. The observed variation across groups in the distribution of ancestry across chromosomes may result from differences in the natural and artificial selective regimens that these populations have experienced. Alternatively, if these breeds underwent strong bottlenecks following admixture, drift could have resulted in rapid fixation of admixed chromosomes before recombination acted to distribute introgressed material. The variation across groups in which chromosomes have biased introgression suggests that differences are not due to chromosomal rearrangements or other barriers to recombination. If there were barriers to recombination on certain chromosomes, those chromosomes would be expected to have more skewed ancestry because recombination could not act to break up ancestral genotypes.

In contrast with the lack of consistent pattern across the autosomes, the X chromosome was the most extreme outlier in 3 groups: recent hybrid cattle, N’Dama cattle, and New World cattle. Indicine ancestry was reduced on the X chromosome compared with the autosomes in all 3 of these groups.

Several genetic characteristics differentiate the X chromosome from autosomes. The population size of the X chromosome is reduced compared with that of autosomes because males only have 1 X chromosome. In addition, apart from the pseudoautosomal region, the X chromosome only undergoes recombination in females. The combination of these 2 facts makes drift a stronger force on the X chromosome than in the autosomes, which could result in differences in apparent admixture among chromosomes. Although the Y chromosome is acrocentric in indicine cattle and submetacentric in taurine cattle, there are no obvious karyotypic differences between the X chromosomes in the 2 groups (Frisch et al. 1997).

Sex-biased introgression may also explain the reduced indicine component on the X chromosomes of the various admixed groups. Using microsatellite markers, MacHugh et al. (1997) found that indicine introgression appeared to be male mediated. If admixed males from an F_1_ generation were preferentially used in backcrosses to a parental line, this practice would decrease the contribution of introgression on the X chromosome relative to the autosomes. As standard breeding practices tend to preserve female offspring in preference to male offspring, this scenario seems unlikely. The pattern could also be influenced by the common breeding practice of using a single bull to inseminate many cows.

Rapid evolution of sex chromosomes has been shown to lead to reproductive isolation among populations (Kitano et al. 2009). However, the lack of biased introgression on the X chromosome in Boran cattle suggests that X chromosome–autosome incompatibilities between taurine and indicine cattle are not responsible for the reduced levels of indicine introgression seen in the X chromosomes of other admixed breeds.

Evidence of indicine ancestry is nearly absent on the X chromosome in New World cattle, with the exception of the recent hybrid individual marked in Figure 3. This absence of X-linked indicine loci is consistent with the hypothesis that New World cattle have been derived from crossing taurine Iberian cattle with admixed western African cattle. This cross would decrease the already reduced introgression on the X chromosome in western African cattle. The near-complete absence of indicine ancestry makes the X chromosome sequences useful for detecting recent indicine introgression in New World cattle.

Applying genome-wide data in conjunction with linkage information affords researchers the ability to more closely examine the patterns and processes of hybridization. There has been rapid development in methods for inferring admixture histories in human populations. In this study, we extended those techniques and applied them to understanding admixture in cattle. By incorporating linkage information and assessing the distribution of ancestry across the genome of admixed groups, we were able to gain a finer-scale understanding of patterns of admixture. We were able to differentiate timing of admixture even among individuals with equal proportions of introgressed ancestry. As genomic data become available for more taxa, these techniques will be able to be widely applied.

## Funding

Graduate Program in Ecology, Evolution, and Behavior at the University of Texas at Austin; Texas EcoLabs; Texas Longhorn Cattleman’s Foundation; National Science Foundation BEACON (Cooperative Agreement DBI–0939454); Extreme Science and Engineering Discovery Environment (XSEDE), National Science Foundation (OCI–1053575).
